# Association between cognitive insight and anxiety among community-dwelling schizophrenia patients: the chain mediating effects of family cohesion and chronotype

**DOI:** 10.3389/fpsyt.2024.1371431

**Published:** 2024-08-19

**Authors:** Meijun Dong, Dongmei Wu, Guoju Mao, Zhangrong Yan

**Affiliations:** ^1^ School of Nursing, Chengdu University of Traditional Chinese Medicine, Chengdu, China; ^2^ Nursing Department, The Clinical Hospital of Chengdu Brain Science Institute, MOE Key Laboratory for Neuroinformation, University of Electronic Science and Technology of China, Chengdu, China

**Keywords:** schizophrenia, cognitive insight, anxiety, family cohesion, chronotype, community, mediating effect

## Abstract

**Objective:**

To investigate the effect of cognitive insight on anxiety in community-dwelling schizophrenia patients and to construct a chain mediating model to determine the underlying mechanisms of the relationship between cognitive insight and anxiety through family cohesion and chronotype.

**Methods:**

The Beck Cognitive Insight Scale, the Chinese version of the Family Adaptability and Cohesion Scale, the Morningness-Eveningness Scale, and the Generalized Anxiety Disorder Scale were used to assess cognitive insight, family cohesion, chronotype, and anxiety in a sample of 785 individuals with schizophrenia living in the community. Spearman correlation analysis, multiple linear regression and Bootstrap methods were employed to analyze the four variables.

**Results:**

Residential region, current employment status, and family economic status impacted anxiety among community-dwelling schizophrenia patients. All variables were associated with each other, but self-certainty was not significantly associated with anxiety. Cognitive insight and self-reflection had direct effects on anxiety, as well as indirect effects on anxiety through the partial mediating effect of chronotype, the suppressing effect of family cohesion, and the chain mediating effect of family cohesion and chronotype.

**Conclusions:**

Family cohesion and chronotype can mediate the relationship between cognitive insight and anxiety. Improving family functioning, promoting emotional relationships within families, and correcting non-circadian sleep patterns can reduce anxiety in community-dwelling schizophrenia patients, with important implications for improving adverse mental health outcomes.

## 1 Introduction

Anxiety symptoms are common throughout the course of schizophrenia, with about 65% of patients with schizophrenia having anxiety symptoms (1), and 38.3% having comorbid anxiety disorders (2). In the field of psychiatry, there is growing concern that anxiety affects the course of psychosis, prognostic outcomes, and individual social functioning. On the one hand, anxiety in early life may induce the onset of schizophrenia in adulthood (3), and severe anxiety symptoms can increase the severity of psychiatric symptoms (such as delusion and illusion) and the possibility of recurrence of schizophrenia (4). On the other hand, anxiety also damages the social function of patients with schizophrenia, and studies have confirmed that schizophrenia patients with social anxiety disorder have more suicide attempts and substance/alcohol abuse (5). Therefore, recognition and treatment of anxiety symptoms are essential for patients with schizophrenia to obtain better clinical outcomes. According to relevant studies, anxiety is related to incomplete cognitive insight (6, 7), and the worse the cognitive insight, the more obvious the anxiety.

Cognitive insight refers to the ability to distance oneself from distorted misconceptions and to use external feedback to evaluate oneself correctly (8). Cognitive insight consists of two elements: self-reflection and self-certainty. Self-reflection helps individuals to better consider and evaluate different views and opinions from the outside world, self-certainty means that individuals are overconfident in the accuracy of their own conclusions and beliefs (8). The relationship between anxiety and cognitive insight has become progressively clearer, with previous studies not only noting that anxiety in psychiatric patients is positively correlated with cognitive insight and self-reflection (9), but suggesting that cognitive insight could predict mental health status in a sample of 420 undergraduate students with subclinical mental illness, implying that the higher scores on cognitive insight were, the more severe the anxiety (10). Also among psychiatric outpatients, rumination, which had similarities to self-reflection (11), could positively predict the prevalence of anxiety (12). Inpatients with psychosis have more severe positive and negative symptoms than those in the community (13), they may experience anxiety at different levels of severity. Thus, the relationship between cognitive insight and anxiety in hospitalized schizophrenia may differ from that in community schizophrenia. Although the relationship between cognitive insight and anxiety has been demonstrated in inpatient schizophrenia, there are few reports in community schizophrenia, and the underlying mechanisms of the relationship are unclear.

Family cohesion refers to the emotional bonds between family members, which is an important indicator of family function. Research has shown that insight negatively predicts family cohesion, with each 1-point increase in relabeling of psychotic experiences in insight being followed by a 0.031-point decrease in family closeness (14). In addition, family cohesion is negatively associated with mental health, with lower family closeness tending to be accompanied by more severe mental health symptoms such as anxiety and depression (15, 16), whereas close family cohesion has a protective effect against emotional distress in schizophrenia, with family cohesion playing the role of mediator of the effects of family therapy on anxiety, and increased family cohesion reducing levels of depression, anxiety and stress in schizophrenia (17). Promoting emotional connection among family members facilitates the alleviation of poor mental health. In summary, this study hypothesizes that family cohesion may be a mediating variable in the relationship between cognitive self-awareness and anxiety.

Chronotype, a potential indicator of circadian rhythm, refers to an individual’s preferences for activity and sleep timing (18). Chronotypes are commonly categorized into three types: morning type, evening type, and intermediate type (19). When an individual’s circadian rhythm is inconsistent with their chronotype, neurocognitive function and higher-order executive functions such as reflection and decision-making are affected (20). For example, daytime sleepiness is significantly associated with cognitive decline (21), and a longitudinal study from China also confirmed that older adults with dysregulated circadian rhythms have a higher risk of cognitive decline (22). In addition, a large body of evidence has summarized the complex relationship between chronotype and anxiety, such as the evening type being associated with anxiety (23), the morning type being associated with anxiety (24), or chronotype not being associated with anxiety (25). Regardless, researchers exploring the relationship between circadian rhythms and anxiety symptoms using clock-genotype combinations based on machine learning methods had found that individuals with circadian rhythm misalignment were at higher risk of severe anxiety symptoms (26). Therefore, we hypothesize that chronotype would be a mediating variable in the relationship between cognitive insight and anxiety.

Family functioning has an impact on an individual’s sleep, emotional bonds between family members have been reported to have an effect on sleep quality and chronotype. Researchers have found that family members have better sleep quality when emotional bonds are stronger (27), and another finding from a study in Taiwan suggested that college freshmen who perceive less support or less emotional care from family members tend to have an evening type schedule (28). Chronotype of schizophrenia in the community who primarily reside in a family environment may be influenced by the emotional bonds of family members, and thus we hypothesized that family cohesion and chronotype would mediate the relationship between cognitive insight and anxiety.

Although the association between cognitive insight and anxiety in psychosis patients is gradually becoming clear, it is questionable whether this association exists in community-dwelling schizophrenia patients, and the mechanism of the relationship between cognitive insight and anxiety is still unclear. The purpose of this study was to analyze the relationship between cognitive insight and anxiety among community-dwelling schizophrenia patients, and to examine the mediating effect of chronotype and family cohesion, which may provide a basis for reducing anxiety and maintaining a healthy psychological state in community schizophrenia patients. The present study made the following hypotheses: 1. cognitive insight in community schizophrenia positively predicts anxiety; 2. family cohesion mediates the relationship between cognitive insight and anxiety; 3. chronotype mediates the relationship between cognitive insight and anxiety, and 4. family cohesion and chronotype chain mediate the relationship between cognitive self-awareness and anxiety.

## 2 Materials and methods

### 2.1 Design and participants

In this study, a convenience sampling method was used to recruit participants from Pengzhou, China, and an online questionnaire survey was used to collect data. A total of 818 community-dwelling patients with schizophrenia participated in the study. A total of 785 valid questionnaires were collected, with an effective rate of 95.97%.

### 2.2 Inclusion criteria and exclusion criteria

The inclusion criteria included the following: (1) Patients diagnosed with schizophrenia by two or more attending psychiatrists and who met the Diagnostic and Statistical Manual of Mental Disorders, Fifth Edition (DSM-5) diagnostic criteria for schizophrenia; (2) Community follow-up patients; and (3) Patients aged between 18 and 60 years. The exclusion criteria were as follows: (1) other mental or neurological diseases, brain developmental disorders, severe trauma, or physical diseases; and (2) a history of drug and alcohol dependence.

Before the start of the survey, all eligible and voluntary participants were informed of the purpose and significance of the study, and informed consent was obtained from the participants before commencing the formal investigation.

### 2.3 Measures

#### 2.3.1 Sociodemographic data

A self-designed questionnaire was used to collect data on the sociodemographic characteristics of community-dwelling schizophrenia patients, including gender, residential region in the past year, marital status, employment status, and household monthly disposable income.

#### 2.3.2 Cognitive insight

The Beck Cognitive Insight Scale (BCIS) is used to measure patients’ abilities to distance themselves from and reassess abnormal beliefs and misconceptions (8). The BCIS has two subscales (self-reflection and self-certainty), with a total of 15 items. The Cognitive Insight composite index score is equal to the self-reflection score minus the self-certainty score, with a higher total score representing better cognitive insight. The Taiwanese version (29) was used in this study, and the Cronbach’s α for self-reflection and self-certainty were 0.70 and 0.72, respectively.

#### 2.3.3 Chronotype

The reduced Morningness-Eveningness Questionnaire (rMEQ) was used to appraise the sleep-wake circadian phase or chronotype (30). Patients with schizophrenia retrospectively assessed their sleep-wake conditions over the past few weeks and selected the closest response. The revised Chinese version of the scale was used in this study, which has good psychometric properties (31). There are 5 items, and the total score ranges from 4 to 25. The higher the overall score, the more evident the trait of waking up and going to bed early becomes, and the less prevalent the trait of staying up and waking up late.

#### 2.3.4 Family cohesion

The Chinese version of the Family Cohesion and Adaptability Scale (FACESII-CV) includes 30 items and is divided into two subscales: family cohesion and family adaptability (32). The scale has good psychometric properties (33). In this study, only the family cohesion subscale was used to assess the emotional connection between family members and schizophrenia patients, and higher scores represented better family cohesion.

#### 2.3.5 Anxiety

The Generalized Anxiety Disorder Scale (GAD-7) is a valid tool developed to screen for anxiety and assess its severity (34). The total score of seven items was summarized to measure anxiety in schizophrenia patients by recalling real-life experiences in the past two weeks. The total score ranged from 0 to 21, and the higher the score was, the higher the level of anxiety. Tong et al. applied the Chinese version and validated it in general hospital outpatients (35).

### 2.4 Statistical analysis

IBM SPSS 24.0 was used to analyze the data. Descriptive statistics were used to analyze the sociodemographic characteristics of the sample, and the Shapiro−Wilk tests for normality for continuous variables. Normally distributed and approximately normally distributed variables are reported as the means ± standard deviations, skewed distributed variables are reported as using medians and quartiles, and categorical variables are reported as frequencies and percentages. The ANOVA test and the nonparametric Kruskal−Wallis test were used to compare two or more continuous variables. The mediation model was adjusted according to the significant variables in the univariate analysis, and the significance was set at p < 0.05. If the significance p-value between a variable and anxiety score is < 0.05, it indicates that this variable may be a covariate and should be excluded from the mediating analysis. Spearman correlation analysis was used to analyze correlations. Stepwise linear regression analysis was used to identify variables that were significantly associated with the dependent variable. PROCESS (36) was used to test the hypothesis that chronotypes and family cohesion mediate the relationship between cognitive insight and anxiety. Finally, the significance of mediating effects was tested using bootstrap sampling with the sample set to 5000 replicates and was considered significant if the bias-corrected bootstrap 95% CI did not include zero. We delete the missing data before statistical analysis, that is, if there is a missing data in a sample, the sample is deleted. Therefore, there is no missing data in this study.

## 3 Results

### 3.1 Sociodemographic characteristics

A total of 785 participants, including 414 (52.7%) men and 371 (47.3%) women, were included in the study. **Table 1** presents descriptive sociodemographic statistics for the community-dwelling schizophrenia patients. Eighty-five point two percent of the patients had lived in rural regions in the past year, 47.6% were married and 70.7% were unemployed, and the household monthly disposable income of 1001-3000 yuan accounted for 51.2% of the sample.

**Table 1:** Status quo and univariate analysis results of anxiety among community-dwelling schizophrenics.

		n (%)	Anxiety		
			M (P_25_,P_75_)	Z	p
Total		785	1 (0,6)		
Gender	male	414 (52.7)	0 (0,5)	2.518	0.113
	female	371 (47.3)	1 (0,6)		
Residential region	rural	669 (85.2)	1 (0,6)	11.256	0.004
	town	86 (11.0)	2 (0,7)		
	city	30 (3.8)	0 (0,0)		
Employment status	student	2 (0.3)	0.5 (0,1)	20.820	0.002
	unemployed	555 (70.7)	2 (0,6)		
	half-time	35 (4.5)	0 (0,2.5)		
	full-time	56 (7.1)	0 (0,5.5)		
	retained	2 (0.3)	1 (0,6)		
	retired	17 (2.2)	0 (0,1)		
	others	118 (15)	0 (0,3)		
Household disposable income (RMB/month)	<1000	270 (34.4)	2 (0,6)	25.620	<0.000
	1001~3000	402 (51.2)	0 (0,6)		
	3001~5000	99 (12.6)	0 (0,3)		
	5001~10000	11 (1.4)	0 (0,7)		
	>10000	3 (0.4)	0 (0,0.5)		
Marital status	unmarried	258 (32.9)	1 (0,6)	6.673	0.154
	married	374 (47.6)	1 (0,5)		
	divorced	127 (16.2)	0 (0,4)		
	widowed	18 (2.3)	0.5 (0,7)		
	others	8 (1.0)	6 (0.25,7)		

**Table 1** also presents the scores for anxiety, as well as differences in demographic characteristics. Residential regions in the last year, employment status, and household monthly disposable income showed significant differences regarding anxiety, so these covariates were excluded in the mediation model.

### 3.2 Correlation analysis

**Table 2** presents the correlations among cognitive insight, anxiety, chronotype, and family cohesion. Cognitive insight, chronotype, and family cohesion scores were (3.30 ± 1.59), (17.36 ± 2.45), (61.93 ± 8.48) respectively. Cognitive insight was negatively correlated with chronotype (r=-0.139, p<0.001), positively correlated with anxiety (r=0.114, p<0.001), and positively correlated with family cohesion (r=0.092, p<0.001). There was no significant correlation between self-certainty and anxiety (r=0.059, p>0.05).

**Table 2:** Correlation of cognitive insight, anxiety, chronotype and family cohesion.

	Mean ± SD	1	2	3	4	5	6
1. cognitive insight	3.30 ± 1.59	1					
2. chronotype	17.36 ± 2.45	-0.139**	1				
3. anxiety	—	0.114**	-0.245**	1			
4. family cohesion	61.93 ± 8.48	0.092**	0.111**	-0.304**	1		
5. self-reflection	10.43 ± 4.79	0.760**	-0.153**	0.096**	0.243**	1	
6. self-certainty	7.12 ± 3.30	0.309**	-0.117**	0.059	0.280**	0.812**	1

**p<0.001.

—, variable conforms to a skewed distribution with no Mean ± SD.

### 3.3 Mediating effect

**Figure 1:**
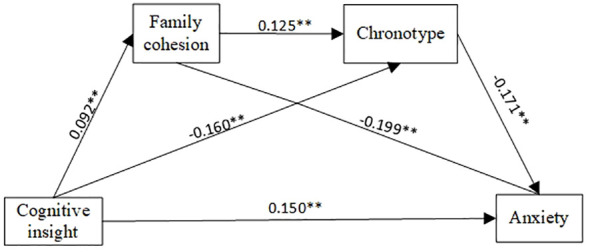
Mediation model of cognitive insight on anxiety.

After controlling for covariates such as residential region in the past year, employment status, and household monthly disposable income, the cognitive insight of community-dwelling schizophrenia patients had a positive predictive effect on anxiety (β=0.161, p<0.001). PROCESS Model 6 was selected to construct the chain mediation model. **Table 3** shows the mediating effect of family cohesion and chronotype on the relationship between cognitive insight and anxiety after controlling for covariates. When family cohesion was used as the dependent variable, cognitive insight had a positive predictive effect (β=0.092, p<0.05). With chronotype as the dependent variable, cognitive insight (β=-0.160, p<0.001) and family cohesion (β=0.125, p<0.001) were significant predictors. With anxiety as the dependent variable, cognitive insight had a positive predictive effect (β=0.150, p<0.001), whereas family cohesion (β=-0.199, p<0.001) and chronotype (β=-0.171, p<0.001) had a negative predictive effect. The model of the mediating effect of cognitive insight on anxiety is shown in **Figure 1**.

**Table 3:** Results of linear regression analysis of cognitive insight, anxiety, chronotype and family cohesion.

	FC				CT				AN			
	*β*	*SE*	95%CI		*β*	*SE*	95%CI		*β*	*SE*	95%CI	
			Lower	Upper			Lower	Upper			Lower	Upper
CI	0.092	0.114	0.087	0.534	-0.160	0.034	-0.222	-0.089	0.150	0.048	0.119	0.309
FC					0.125	0.011	0.015	0.057	-0.199	0.015	-0.114	-0.055
CT									-0.171	0.050	-0.350	-0.152
R	0.324				0.252				0.368			
R^2^	0.101				0.064				0.135			
ΔF	7.409*				14.999**				28.694**			

CI, cognitive insight; FC, family cohesion; CT, chronotype; AN, anxiety; SE, standard error; *p<0.05; **p<0.001.

The results of the Bootstrap test are shown in **Table 4**. Both the direct effect and the total effect were significant, and there was a partial mediating effect. The mediating effects of the three pathways were significant, and there was a chain mediating effect; that is, cognitive insight not only directly affected anxiety but also indirectly affected anxiety through family cohesion and chronotype. Furthermore, it was found that family cohesion had a suppressing effect on the relationship between cognitive insight and anxiety because the effect values of path 1 and path 3 were negative, and the direct effect was positive. In addition, compared with the chain mediating effect (-0.003) and the suppressing effect mediated by family cohesion (-0.026), chronotype had a larger mediating effect (0.039).

**Table 4:** Results of the Bootstrap test of the mediating effect of cognitive insight on anxiety.

	Path	Effect	SE	95%CI	
				Lower	Upper
total effect	CI→AN	0.224	0.050	0.127	0.321
direct effect	CI→AN	0.214	0.049	0.119	0.309
indirect effect	CI→FC→AN	-0.026	0.012	-0.050	-0.005
	CI→CT→AN	0.039	0.013	0.017	0.068
	CI→FC→CT→AN	-0.003	0.002	-0.007	-0.001

CI, cognitive insight; FC, family cohesion; CT, chronotype; AN, anxiety; SE, standard error,

The symbol "→" means paths, and refers to the direction of the relationship between the two variables.

**Figure 2:**
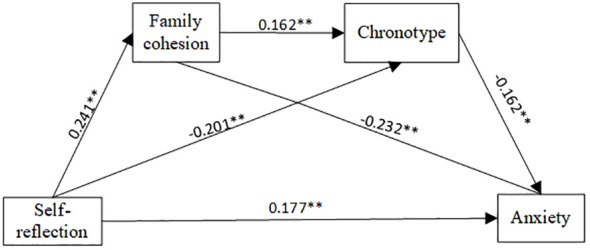
Mediation model of Self-reflection on anxiety.

Subsequently, the chain mediating effect of self-reflection on anxiety was verified. The results showed that self-reflection significantly predicted anxiety (β=0.150, p<0.001). When family cohesion and chronotype were included in the linear regression equation, after controlling for covariates, self-reflection positively predicted family cohesion (β=0.241, p<0.001); self-reflection (β=-0.201, p<0.001) and family cohesion (β=0.162, p<0.001) both influenced chronotype; self-reflection, and chronotype and family cohesion predicted anxiety ((β=0.177, p<0.001), (β=-0.162, p<0.001) and (β=-0.232, p<0.001), respectively). The chain mediating effect of family cohesion and chronotype in community-dwelling schizophrenia patients is shown in **Table 5**, **Figure 2**.

**Table 5:** Results of linear regression analysis of self-reflection, anxiety, chronotype and family cohesion.

	FC				CT				AN				
	*β*	*SE*	95%CI		*β*	*SE*	95%CI		*β*	*SE*	95%CI	
			Lower	Upper			Lower	Upper			Lower	Upper
SR	0.241	0.059	0.311	0.541	-0.201	0.018	-0.139	-0.067	0.177	0.026	0.081	0.184
FC					0.162	0.011	0.026	0.068	-0.232	0.015	-0.129	-0.068
CT									-0.162	0.051	-0.336	-0.137
R	0.393				0.275				0.376			
R^2^	0.150				0.076				0.141			
ΔF	52.940*				20.329**				30.753**			

SR, self-reflection; FC, family cohesion; CT, chronotype; AN, anxiety; SE, standard error; *p<0.05; **p<0.001.

When family cohesion and chronotype were included in the regression, the direct effect (0.133) was greater than the total effect (0.110) because the negative value of the suppressing effect offset the positive value of the mediating effect. The bootstrap test was used to analyze the significance of the mediating effect, as shown in **Table 6** (path 1 (effect=-0.042, 95% CI [-0.059, -0.026]), path 2 (effect=0.024, 95% CI [0.011, 0.041]), and path 3 [effect=-0.005, 95% CI [-0.009, -0.002)].

**Table 6:** Results of the Bootstrap test of the mediating effect of self-reflection on anxiety.

	Path	Effect	SE	95%CI	
				Lower	Upper
total effect	SR→AN	0.110	0.026	0.059	0.1616
direct effect	SR→AN	0.133	0.026	0.0807	0.1843
indirect effect	SR→FC→AN	-0.042	0.008	-0.059	-0.026
	SR→CT→AN	0.024	0.008	0.011	0.041
	SR→FC→CT→AN	-0.005	0.002	-0.009	-0.002

SR, self-reflection; FC, family cohesion; CT, chronotype; AN, anxiety; SE, standard error.

The symbol "→" means paths, and refers to the direction of the relationship between the two variables.

## 4 Discussion

This study explores whether there are differences in the sociodemographic characteristics of community-dwelling schizophrenia patients regarding anxiety, and a chain mediation model is constructed to explore the underlying mechanisms of family cohesion and chronotype in the relationship between cognitive insight and anxiety.

We found that community-dwelling schizophrenics with severe anxiety were characterized by the following characteristics: living in a town community, currently unemployed status, and having <1000(RMB/month) household disposable income. This is consistent with previous findings that urban residence is a risk factor for anxiety, with living in an urban area increases the risk of anxiety by 1.24 times compared with living in other areas (37, 38), which may be related to the fact that people with schizophrenia in urban neighborhoods have a poorer subjective sense of safety leading to anxiety (39), and the relative scarcity of natural outdoor environments in cities can further increase the risk of anxiety prevalence (40). In addition, lower perceived social support is associated with more anxiety symptoms (41), such as unemployed status and low average monthly income. Therefore, it is recommended to seek professional medical services for schizophrenics, improve the community living environment, and encourage them to participate in social work to obtain social support to help maintain mental health.

Correlations among anxiety, cognitive insight, family cohesion, and chronotype were demonstrated. This is consistent with previous research showing that psychosis patients with higher cognitive insight or self-reflection are more prone to anxiety (42–44). Poor family cohesion leads to sleep problems (45), and family emotional bonds promote the formation of good sleep habits in individuals (46) and improve circadian rhythm disorders. Strong family bonds and circadian sleep patterns also contribute to lower levels of anxiety (47–51).

The results of the chain mediating effect of cognitive insight on anxiety showed that cognitive insight could directly predict anxiety, and it could also predict anxiety through the partial mediating effects of family cohesion and chronotype. Family cohesion and chronotype played a chain mediating role in the effect of cognitive insight on anxiety.

The direct effect of cognitive insight/self-reflection on anxiety has been demonstrated in previous studies, which supports Hypothesis 1. Self-reflection was found to predict anxiety in adolescent athletes, such as cognitive anxiety and somatic anxiety (52). Among 175 patients with trait anxiety, it was found that cognitive insight/self-reflection was positively correlated with negative metacognition, and anxious patients were more likely to have negative metacognition skills (53). However, other results are contrary to our findings; for example, there was no correlation between self-reflection and anxiety symptoms in outpatients with schizophrenia (54); self-reflection was negatively correlated with social anxiety in college students recruited by a convenience sampling method (55), which mean that individuals with low self-reflection may have more anxiety, and self-reflection is a protective factor against social anxiety. Regardless, these explanations help us to understand why self-reflection is associated with anxiety.

Cognitive insight/self-reflection also affected anxiety through the suppressing effect of the indirect pathway of family cohesion, which supports Hypothesis 2. In a 1-year longitudinal study of individuals with schizophrenia spectrum disorders, higher cognitive insight was found to be significantly associated with lower life satisfaction (56) and worse mental health outcomes (10). Others also found that higher self-reflection means greater vulnerability to emotional distress, such as suicidal ideation, but family cohesion is a protective factor against suicidal ideation (57). It may be that patients with high self-reflection often repeatedly consider the views and ideas of others, especially schizophrenia patients, who often rely heavily on their family members for support. Therefore, family members participate in more communication, information sharing, and group activities to promote family cohesion and form a group-centered problem-solving method. Many studies have reported the direct or indirect effects of family cohesion on emotions, for example, higher family cohesion is negatively associated with anger (58). Family cohesion is a moderator of resilience and negative emotions, while resilience has a greater effect on alleviating depressive symptoms (59), and family cohesion enhances the negative correlation between parental emotional socialization behaviors and adolescent emotional disorders (60). Therefore, research advocates that developing high-quality and more united family emotional bonds is necessary to maintain mental health because family cohesion is a potential protective factor against anxiety.

The results suggest that cognitive insight affects anxiety through a partial mediation of the indirect pathway of chronotype, which supports Hypothesis 3. In other words, when cognitive insight/self-reflection gets higher, patients are more likely to have chronotype changes or circadian rhythm misalignment and more likely to go to bed and wake up later. A possible reason is that schizophrenics tend to overthink their own abnormal experiences and the guidance feedback of others, leading to additional self-reflection (61), which in turn affects chronotype through self-control. It has been shown previously that metacognition predicts self-control (62), that higher self-reflection is associated with better self-control, and self-control is significantly associated with chronotype, with individuals with lower self-control being more likely to have an evening chronotype (63). In addition, self-control also mediates the relationship between chronotype and temporal perspective. Individuals with morning chronotypes have a future-oriented temporal perspective due to self-control, whereas individuals with evening chronotypes are more present-oriented due to poor self-control (64). This conclusion is indirectly corroborated by the bidirectional association theory of sleep and wake behaviors, which suggests that later bedtimes and wake times lead to later bedtimes and wake times the next day, whereas earlier bedtimes and wake times predicted higher positive emotions the next day (65), which is consistent with the conclusion of this study on the relationship between chronotype and mood dysregulation. Therefore, it is suggested that from the perspective of sleep time, building a normal circadian rhythm of work and rest habits is beneficial n relieving anxiety.

This study also contributed to providing support for the chain mediating effect of family cohesion and chronotype on the relationship between cognitive insight and anxiety, which supports Hypothesis 4. In the chain mediating model, chronotype played a mediating role between family cohesion and anxiety, that is, family cohesion could reduce anxiety by maintaining normal sleep rhythms. Consistent with our findings, Palimaru et al. found that enhanced family cohesion had a direct effect on reducing depressive and anxiety symptoms and an indirect effect through a reduction in sleep disturbance, such as erratic sleep-wake behavior (66). In a large sample of Norwegian adolescents, those living in families with separated parents had a 1.81 times higher risk of a delayed sleep phase, and a delayed sleep phase was positively associated with anxiety (67), that is, adolescents with poor emotional family bonds were more likely to have sleep problems, and the more severe the sleep problems were, the more severe the anxiety. In conclusion, family cohesion and sleep play important positive roles in maintaining physical and mental health.

In addition, the mediating analysis of the relationship between cognitive insight and anxiety shows that the indirect effect of chronotype accounts for 17.03% of the total effect, while family cohesion is 11.61%, which indicates that chronotype has a greater impact on cognitive insight and anxiety than family cohesion. Whereas, the mediating analysis of the relationship between self-reflection and anxiety showed that the indirect effect of time type accounts for 21.82% of the total effect, while family cohesion is 38.18%, which indicates that family cohesion has a greater impact on self-reflection and anxiety. These results all suggest the importance of mediating variables such as family cohesion and chronotype in improving anxiety in schizophrenia. However, more and more mechanisms remain to be explored in depth.

The current study explored the association between cognitive insight and anxiety and analyzed the chain mediating role of family cohesion and chronotype in this relationship. These results reveal the specific mechanism of the effect of cognitive insight on anxiety and provide a more scientific and comprehensive basis for improving the adverse mental health outcomes of community-dwelling schizophrenia patients.

## 5 Strengths and limitations

This study was part of a large follow-up study, and the follow-up data during the COVID-19 period better reflected the protective effect of family function against psychotic anxiety symptoms, which provided inspiration of future mental health promotion for patients with psychosis.

However, there are still limitations. First, this study only included schizophrenia patients in Pengzhou city, and the results may not be generalizable to the whole country. Second, data collection was based on self-reported online questionnaires, which may have bias. Third, the screening of mental or neurological diseases, brain developmental disorders, severe trauma, or physical diseases is mainly through the observation and physical examination of two or more attending psychiatrists, rather than CT examination, nuclear magnetic resonance, blood test and other objective indicators. The measurement of objective indicators should be considered in the future research. In addition, our data analysis does not take into account the impact of confounding factors such as the severity of schizophrenia symptoms, medication use, or access to mental health services. Future studies should be carefully described and controlled in all aspects.

## 6 Conclusion

This study preliminarily explored the relationship between cognitive insight and anxiety in community-dwelling schizophrenia patients by constructing a chain mediation model. Family cohesion and chronotype were found to be mediating variables of the relationship between cognitive insight and anxiety. This study suggests that enhancing emotional bonds among family members and maintaining a normal rhythm rest time can help to reduce anxiety. Future research can formulate effective treatment plans for patients’ mental health from the perspective of internal family relations combined with circadian rhythm.

## Data Availability

The raw data supporting the conclusions of this article will be made available by the authors, without undue reservation.
